# Misconceptions on COVID-19 Risk Among Ugandan Men: Results From a Rapid Exploratory Survey, April 2020

**DOI:** 10.3389/fpubh.2020.00416

**Published:** 2020-07-28

**Authors:** Keneth Iceland Kasozi, Ewan MacLeod, Fred Ssempijja, Michael W. Mahero, Kevin Matama, Grace Henry Musoke, Kevin Bardosh, Robinson Ssebuufu, Florence Wakoko-Studstil, Isaac Echoru, Emmanuel Tiyo Ayikobua, Regan Mujinya, Grace Nambuya, Hope Onohuean, Gerald Zirintunda, Justine Ekou, Susan Christina Welburn

**Affiliations:** ^1^Infection Medicine, Deanery of Biomedical Sciences, College of Medicine and Veterinary Medicine, The University of Edinburgh, Edinburgh, United Kingdom; ^2^Faculty of Biomedical Sciences, Kampala International University Western Campus, Bushenyi, Uganda; ^3^Department of Veterinary Population Medicine, College of Veterinary Medicine, University of Minnesota, St. Paul, MN, United States; ^4^School of Pharmacy, Kampala International University Western Campus, Kampala, Uganda; ^5^Faculty of Science and Technology, Cavendish University, Kampala, Uganda; ^6^Center for One Health Research, School of Public Health, University of Washington, Seattle, WA, United States; ^7^Faculty of Clinical Medicine and Dentistry, Kampala International University Teaching Hospital, Bushenyi, Uganda; ^8^Department of Criminal Justice & Sociology, College of Letters and Sciences, Columbus State University, Columbus, GA, United States; ^9^Department of Anatomy, School of Medicine, Kabale University, Kabale, Uganda; ^10^Department of Physiology, School of Health Sciences, Soroti University, Soroti, Uganda; ^11^Faculty of Agriculture and Animal Sciences, Busitema University, Tororo, Uganda; ^12^Zhejiang University-University of Edinburgh Institute, Zhejiang University School of Medicine, International Campus, Zhejiang University, Haining, China

**Keywords:** COVID-19, COVID-19 response in Africa, impact of COVID-19 in Uganda, myths about COVID-19, United Nations emergency appeal response to COVID-19, gender matters in COVID-19 response, impact of COVID-19 in children, efforts to mitigate and adapt to COVID-19

## Abstract

**Background:** Transmission of COVID-19 in developing countries is expected to surpass that in developed countries; however, information on community perceptions of this new disease is scarce. The aim of the study was to identify possible misconceptions among males and females toward COVID-19 in Uganda using a rapid online survey distributed *via* social media.

**Methods:** A cross-sectional survey carried out in early April 2020 was conducted with 161 Ugandans, who purposively participated in the online questionnaire that assessed understandings of COVID-19 risk and infection. Sixty-four percent of respondents were male and 36% were female.

**Results:** We found significant divergences of opinion on gendered susceptibility to COVID-19. Most female respondents considered infection risk, symptoms, severe signs, and death to be equally distributed between genders. In contrast, male respondents believed they were more at risk of infection, severe symptoms, severe signs, and death (52.7 vs. 30.6%, RR = 1.79, 95% CI: 1.14–2.8). Most women did not share this perception and disagreed that males were at higher risk of infection (by a factor of three), symptoms (79% disagree), severe signs (71%, disagree), and death (70.2% disagree). Overall, most respondents considered children less vulnerable (OR = 1.12, 95% CI: 0.55–2.2) to COVID-19 than adults, that children present with less symptoms (OR = 1.57, 95% CI: 0.77–3.19), and that there would be less mortality in children (OR = 0.92, 95% CI: 0.41–1.88). Of female respondents, 76.4% considered mortality from COVID-19 to be different between the young and the elderly (RR = 1.7, 95% CI: 1.01–2.92) and 92.7% believed young adults would show fewer signs than the elderly, and 71.4% agreed that elderly COVID-19 patients would show more severe signs than the young (OR = 2.2, 95% CI: 1.4, 4.8). While respondents considered that all races were susceptible to the signs and symptoms of infection as well as death from COVID-19, they considered mortality would be highest among white people from Europe and the USA. Some respondents (mostly male 33/102, 32.4%) considered COVID-19 to be a “disease of whites” (30.2%).

**Conclusion:** The WHO has identified women and children in rural communities as vulnerable persons who should be given more attention in the COVID-19 national response programs across Africa; however, our study has found that men in Uganda perceive themselves to be at greater risk and that these contradictory perceptions (including the association of COVID-19 with “the white” race) suggest an important discrepancy in the communication of *who* is most vulnerable and *why*. Further research is urgently needed to validate and expand the results of this small exploratory study.

## Introduction

The new pandemic caused by the severe acute respiratory syndrome coronavirus 2 (SARS-CoV-2) emerged in late 2019, disrupting health systems and critical care, even within the most developed heath systems and economies (1). As of April 22nd, 2020, 2.5 million confirmed cases of COVID-19 have been reported globally (2). Sustained community transmission is expected in low- and middle- income countries (LMICs) where COVID-19 containment strategies continue to be a challenge (3). Extreme mitigation strategies have been put in place in many countries to control COVID-19, to reduce disease transmission and to avoid overburdening healthcare systems including mass lockdowns, curfews, and social distancing measures (4). SARS-CoV-2 and interventions to reduce transmission are negatively impacting already impoverished communities in LMICs and will test heath systems that have little capacity for the management of high dependency patients, or sufficient PPE to protect health workers (5). Interventions will have long-lasting detrimental impacts on LMIC economies, and, in the absence of reliable and efficient tools for early detection of infected and exposed individuals, are likely to extend beyond 2020/21 including in Africa (6).

Africa is vulnerable to being overwhelmed by COVID-19. The World Health Organization (WHO) Director General Dr. Tedros Ghebreyesus, stated that the greatest concern was COVID-19 transmission in countries with weaker health systems than in developed nations (7). On Apr 17, 2020, the WHO estimated 10 million cases of COVID-19 spreading rapidly across Africa and up to 3 million deaths within 6 months (8). Cases are expected to rise quickly due to a chronic lack of testing, lack of personal protective equipment (PPE), and poor patient care facilities of basic equipment to contain the pandemic, such as PPE (9, 10). The ability to contain COVID-19 will depend on the success of social distancing and the ability to diagnose, isolate, and treat cases (11).

Case finding and reporting for COVID-19 in Africa is making less than ideal progress. Data from the African Centers for Disease Control (CDC) shows that while risk of importation of COVID-19 to Africa was lower than that to Europe (1 vs. 11%), response and reaction capacity are also lower; the latter being intrinsically linked to individual country wealth and resources for detection, prevention, and control (12). In late March, Africa had reported 41 local transmissions and only 9 imported cases, by 7 April 2020, 9,888 of 9,971 cases (99.2%) were community acquired with only 83/9,971 cases being imported. As of 18 April 2020, Africa had reported 1,000 deaths with COVID-19 and more than 19,800 cases in 52 out of 54 countries on the African continent (13). With travel restrictions in place, all cases of COVID-19 are considered community acquired (14). While many African nations have employed lessons learned from Ebola (15). COVID-19 is far more challenging to manage. Quantifying the pandemic growth across the African sub-continent and assessing the impact of interventions put in place will be compromised by the lack of diagnostic capacity (16).

Across East Africa, in April 2020, countries lack a coordinated response against COVID-19. While the WHO/AFRO are making strong recommendations, many governments are taking their own approach. The president of Tanzania encouraged people to “pray for 3 days” against COVID-19 and has not imposed any movement restrictions—places of worship remain open (17). In Kenya, only a partial lockdown is in place in major cities and many are not prepared for a total lockdown of the country (18, 19). In Uganda and Rwanda more extreme actions have been taken with total national lockdowns that have involved closure of all non-essential businesses, public transport, and the closure of schools and universities. Only local food stores, supermarkets, medical, and veterinary supplies are exempt and for these services a mandatory curfew has been imposed. No motor vehicles/motorists are permitted to use public roads unless they are listed among essential service providers authorized by the Office of the President (20–22). Countries not abiding with the social distancing guidelines as recommended by the WHO are putting neighboring countries at risk and compromising health security within the East African Community (EAC). The EAC continues to show a disorganized response against COVID-19 and this high level of disorganization has created confusion in the general public as to what is “true” and “false” COVID-19 information. Rumors and misinformation have spread widely within the community and the media; for example, a religious leader in Uganda claimed that there was no COVID-19 in Uganda and stated that it was just “simple flu” and an individual at Kampala City Council Football Club falsely claimed that Uganda had lost a patient to COVID-19; these culprits are currently in jail awaiting trial for spreading false information (23, 24). Risks and assumptions are causing disharmony in communities, fearful of risk of infection and of the economic consequences of government interventions.

To date, COVID-19 cases and deaths have been greatest in Europe, the Americas, the Western Pacific, the Eastern Mediterranean, South East Asia, and Africa, but the situation is fluid and will change as COVID-19 impacts new regions (25). While most COVID-19 patients have been of European, Asian, and African descent, data from the USA indicates that while African-Americans are at equal risk of infection with SARS-CoV-2, they are at higher risk of severe COVID-19 complications and death. In Illinois, USA, African Americans accounted for 29% of confirmed cases and 41% of deaths, yet comprise 15% of the state's population (26). Similar trends have been observed in Michigan and Wisconsin, USA (27). This is unlikely to have a basis in racial susceptibility but is more likely due to a vulnerability to infection and lack of access to quality medical care—it's not about race but about racism and poverty (28). These observations are of concern for developing countries in Africa and Latin America (29).

The prognosis for COVID-19 infection in Asia and Europe appears to be influenced by sex (being male), pre-existing health conditions (diabetes, cancers, and cardiovascular disease), and age (average age 81 years) (30); other risk factors may include air pollution and smoking (31). Reports released by the Chinese Center for Disease Control and Prevention show that men are more at risk than women (32). Sex differences in males and females in China are supported by the relatively higher antibodies titer generated in females against COVID-19 (33). Reports from Italy showed no significant differences between males and females infected with COVID-19 (34). Older males continue to be disproportionally affected by COVID-19 (35).

In young people (<18 years), reported COVID-19 infections, hospitalization, and death are low (36). COVID-19 generally presents with milder symptoms in children than in adults (3, 7), but the evidence-base is unclear. This does not mean children are immune, and children are considered important asymptomatic carriers able to facilitate SARS-CoV-2 transmission within households (37).

While most COVID-19 infections are mild (with <20% of cases being severe to critical) (38), communities of heavy smokers or those with lung function impairments are believed to be particularly susceptible to complications (39). The WHO has stated that women in Africa are most likely to die from COVID-19 due to sex inequity, chronic poverty among women, weak economic capacity, sexual, and gender-based violence (40). The elderly are more vulnerable to coronavirus (41) and underlying non-communicable diseases that are pervasive in Africa will predispose individuals to complications from SARS-CoV-2 infection.

This study aimed to identify perceptions of COVID-19 risk among Ugandans in order to identify novel strategies to guide the national COVID-19 Task Force (nCTF) to improve, control, and prevent COVID-19 infections.

## Methods

This was a cross sectional study conducted with 161 Ugandan respondents in the second week of April 2020. During this period, COVID-19 infections started to increase in East Africa and Uganda was placed in total lockdown (March–May 2020). A pre-tested online questionnaire using Q-survey® (https://www.qsurvey.qa/home/en) was administered with study participants through online resources i.e., email, Facebook, Twitter, WhatsApp, and Viber. Only Ugandans were included in the study while international residents were excluded from the study, using phone IP addresses, which were automatically generated by the Q survey®. Study participants were encouraged to share the link to the questionnaire with family members and friends to enhance data collection using the same social media platforms. Financial challenges in this period implied that the response rate was low since a majority of Ugandans use prepaid mobile internet connection. The questionnaire was designed using major trending informal statements on COVID-19 to assess the knowledge and perceptions of COVID-19 (Supplementary File 1). Completing the questionnaire in full was not a mandatory requirement and some questions could be skipped. Metadata was collected and only study participants whose current location was in Uganda was preserved for statistical analysis.

### Statistical Analysis

Data was exported from Q survey in MS Excel and univariate statistics were conducted using WinPEPI® and significance was reported at a 95% confidence interval using questions which were asked to both males and females in Uganda.

## Results

### COVID-19 Perceptions Amongst Male and Female Ugandans

Of 161 Ugandan respondents, 64% (*n* = 103, 95% CI: 56.3–71.1) were male and 36% were female (*n* = 58, 95% CI: 28.9–43.7). Most were between the ages of 18–30 years (70.5%, 110/161) (Table 1). A total of 52.7% of male respondents (54/102) considered males to be more vulnerable to infection with COVID-19 while 70.4% of female respondents (38/54) did not consider males to be more vulnerable; a significant difference between the groups (RR = 1.79, 95% CI: 1.14–2.8). Most female respondents considered infection risk to be equally distributed between genders and there was no significant difference in this perception between male and female respondents.

**Table 1:** Description of the study participants in Uganda.

**Variables**		**Statistic**	**Percentage**	**95% CI**
Sex	Male	103^a^	64.0	56.3–71.1
	Female	58^a^	36.0	28.9–43.7
Total		161^a^	100	98.2–100
Age	18–30	110^a^	70.5	60.8–75.2
	31–55	46^a^	28.5	22.0–35.9
	Undeclared	05^a^	3.1	1.2–6.8
Total		161^a^	100	98.2–100
Descriptive of age (years)				
	Mean	28.2^b^		27.1–29.2
	Median	27.0^b^		25.0–29.0
	Minimum	18.0^b^		
	Maximum	55.0^b^		
	25th Percentile	23.0^b^		
	75th Percentile	31.8^b^		
	SEM	0.6^b^		

a *Frequency*;

b *counts. 95% CI, 95% confidence intervals*.

More male (52.5%, 52/99, 95% CI: 42.7–62.2) respondents believed they were more likely to die from COVID-19 than females (29.6%, 16/54, 95% CI: 18.6–42.8); however 70.2% (40/57) of females disagreed with them on who is more likely to die. Females were half as convinced that men were more likely to die than the male respondents (RR = 1.76, 95% CI: 1.1–2.7). There was no significant difference between the groups of male and female respondents on whether both sexes were equally at risk of death (RR = 0.8, 95% CI: 0.7–1.0). The majority of males (41.4%, 41/99) believed that males present with more severe signs; however, females (71.4%, 40/56) were over two times more likely to disagree with this statement. Female respondents (79%, 45/57) disagreed with the statement that symptoms presented more in males (OR = 1.8, 95% CI: 0.8–4.2). Most female respondents agreed that both sexes showed equal signs for COVID-19 (46/56) however no significant differences were found in males and females (RR = 0.9, 95% CI: 0.8–1.1). The majority of female respondents (*n* = 40/56, 71.4%, 95% CI: 58.6–82.1) did not think males present with more severe signs of COVID-19 than women, although no significant differences were found (RR = 1.5, 95% CI: 0.9–2.3). Most study participants indicated that both sexes shared the same symptoms (Table 2).

**Table 2:** Frequency and percentage of respondents and their risk estimates on signs and symptoms of COVID-19 amongst male and female Ugandans.

**Parameter compared to females**	**Variables**	**Frequency (%) of study participation response**			**Risk estimates (95% CI)**		
		**Male**	**Female**	**Total**	**aR**	**RR**	**OR**
Age (years)	18–30	65 (65.0)	45 (80.4)	110 (70.5)	−15.4 (−30.7,0.0)	0.9 (0.7–1.0)	0.5 (0.2–1.0)
	31–55	35 (35.0)	11 (19.6)	46 (29.5)			
	Total	100 (100)	56 (100)	156 (100)			
Males more vulnerable	Yes	54 (52.9)	16 (29.6)	70 (44.9)	23.3 (6.3–40.3)	1.8 (1.1–2.8)	2.7 (1.3–5.5)
	No	48 (47.1)	38 (70.4)	86 (55.1)			
	Total	102 (100)	54 (100)	156 (100)			
Equal risk to infection	Yes	68 (67.3)	44 (77.2)	112 (70.9)	−9.9 (−25.5–5.7)	0.9 (0.7–1.1)	0.6 (0.3–1.3)
	No	33 (32.7)	13 (22.8)	46 (29.1)			
	Total	101 (100)	57 (100)	158 (100)			
Males more likely to die	Yes	52 (52.5)	17 (29.8)	87 (55.8)	22.7 (5.9–39.5)	1.8 (1.1–2.7)	2.6 (1.3–5.3)
	No	47 (47.5)	40 (70.2)	69 (44.2)			
	Total	99 (100)	57 (100)	156 (100)			
Both sexes equally die	Yes	61 (59.8)	42 (73.7)	103 (64.8)	−13.9 (−30.1–2.4)	0.8 (0.7–1.0)	0.5 (0.3–1.1)
	No	41 (40.2)	15 (26.3)	56 (35.2)			
	Total	102 (100)	57 (100)	159 (100)			
Males have more signs	Yes	32 (32.3)	12 (21.1)	44 (28.2)	11.3 (−4.1–26.7)	1.5 (0.9–2.7)	1.8 (0.8–4.2)
	No	67 (67.7)	45 (78.9)	112 (71.8)			
	Total	99 (100)	57 (100)	156 (100)			
Males show equal signs	Yes	78 (76.5)	46 (82.1)	124 (78.5)	−5.7 (−20.0–8.7)	0.9 (0.8–1.1)	0.7 (0.3–1.6)
	No	24 (23.5)	10 (17.9)	34 (21.5)			
	Total	102 (100)	56 (100)	158 (100)			
Males with more severe signs	Yes	41 (41.4)	16 (28.6)	57 (36.8)	12.8 (−3.9–29.5)	1.5 (0.9–2.3)	1.8 (0.9–3.6)
	No	58 (58.6)	40 (71.4)	98 (63.2)			
	Total	99 (100)	56 (100)	155 (100)			
Both sexes equal to suffer signs	Yes	69 (68.3)	39 (69.6)	108 (68.8)	−1.3 (−17.8, 15.1)	1.0 (0.8–1.2)	0.9 (0.5–1.9)
	No	32 (31.7)	17 (30.4)	49 (31.2)			
	Total	101 (100)	56 (100)	157 (100)			

*Risk estimates conducted with males being the reference category; aR, attributable risk; RR, relative risk; OR, odds ratio; 95% CI, 95% confidence interval*.

### Perceptions on COVID-19 Toward Children and Young Adults Amongst Ugandans

Overall, most respondents, 53.5%, 95% CI: 45.7–61.2 (84/157) considered children to be less vulnerable than adults; no significant differences were found between males and females (RR = 1.1, 95% CI: 0.8–1.5). Similar observations were found on participant responses for children being as equally vulnerable as adults. Most respondents considered mortalities to be less in children (male = 63.7%, 65/102, females = 55.4%, 31/56); however, no significant differences were found among those who disagreed with this perception (RR = 1.15, 95% CI: 0.9, 1.5). In addition, most respondents considered children to present with fewer signs of COVID-19 (82/158), and that the signs of infection are not the same in children (77/156), being less severe than in adults (80/155); there were no significant differences between males and females in these responses (Table 3).

**Table 3:** Descriptive statistics and risk estimates on perceptions of COVID-19 in children and adults amongst male and female Ugandans.

**Parameter compared to adults**	**Variables**	**Frequency (%) of study participation response**			**Risk estimates (95% CI)**		
		**Male**	**Female**	**Total**	**aR**	**RR**	**OR**
Children more vulnerable	Yes	48 (47.5)	25 (44.6)	73 (46.5)	2.9 (−14.8–20.5)	1.1 (0.8–1.5)	1.1 (0.6–2.2)
	No	53 (52.5)	31 (55.4)	84 (53.5)			
	Total	101 (100)	56 (100)	157 (100)			
Children equally vulnerable	Yes	41 (40.2)	20 (36.4)	61 (38.9)	3.8 (−10.9–18.6)	1.1 (0.7–1.7)	1.2 (0.6–2.5)
	No	61 (59.8)	35 (63.6)	96 (61.1)			
	Total	102 (100)	55 (100)	157 (100)			
Children die less	Yes	65 (63.7)	31 (55.4)	96 (60.8)	8.4 (−9.0–25.8)	1.2 (0.9–1.5)	1.4 (0.7–2.9)
	No	37 (36.3)	25 (44.6)	62 (39.2)			
	Total	102 (100)	56 (100)	158 (100)			
Children die equally	Yes	44 (44.4)	26 (46.4)	70 (45.2)	−2.0 (−19.7–15.7)	1.0 (0.−1.4)	0.9 (0.4–1.9)
	No	55 (55.6)	30 (30.0)	85 (54.8)			
	Total	99 (100)	56 (100)	155 (100)			
Children less signs	Yes	57 (55.9)	25 (44.6)	82 (51.9)	11.2 (−6.3–28.8)	1.3 (0.9–1.8)	1.6 (0.8–3.2)
	No	45 (44.1)	31 (55.4)	76 (48.1)			
	Total	102 (100)	56 (100)	158 (100)			
Children equal signs	Yes	52 (52)	27 (48.2)	79 (50.6)	3.8 (−14.0–21.5)	1.1 (0.8–1.5)	1.2 (0.6–2.4)
	No	48 (48)	29 (51.8)	77 (49.4)			
	Total	100 (100)	56 (100)	156 (100)			
Children less severe signs	Yes	55 (55)	25 (45.5)	80 (51.6)	9.5 (−8.2–27.3)	1.2 (0.9–1.7)	1.5 (0.7–3.0)
	No	45 (45)	30 (54.5)	75 (48.4)			
	Total	100 (100)	55 (100)	155 (100)			
Children equally severe signs	Yes	48 (48)	28 (50)	76 (76)	−2.0 (−19.7–15.7)	1.0 (0.7–1.3)	0.9 (0.5–1.9)
	No	52 (52)	28 (50)	80 (80)			
	Total	100 (100)	56 (100)	156 (100)			

*Risk estimates conducted with males being the reference category; aR, attributable risk; RR, relative risk; OR, odds ratio; 95% CI, 95% confidence interval*.

### Perceptions on COVID-19 Risks Among the Elderly

A significantly large proportion of female respondents (76.4%, 42/55) (compared to male respondents 59.4%, 60/101) did not believe mortality rates to be equal in the young and elderly (RR = 1.7, 95% CI: 1.0–2.9). Amongst those who believed that young adults show fewer signs than the elderly, a majority (92.7%, 51/55) of female respondents believed this to be true compared to 84.2% (85/101) of males. Furthermore, a majority of female respondents (71.4%, 40/56)—compared to only 53.1% (52/98) of male respondents agreed that elderly COVID-19 patients would show more severe signs than the young (OR = 2.2, 95% CI: 1.4, 4.8) as shown in Table 4. Among those who believed that young adults are more likely to die of COVID-19 than the elderly (19/156), 16% were males while 5.4% were females and there was no significant difference between them.

**Table 4:** Perceptions on COVID-19 presentation in young adults and the elderly in Ugandans.

**Parameter as compared to the elderly**	**Variable**	**Frequency (%) of study participation response**			**Risk estimates (95% CI)**		
		**Male**	**Female**	**Total**	**aR**	**RR**	**OR**
Young adults are more likely to die	Yes	16 (16.0)	3 (5.4)	19 (12.2)	10.6 (0.0–21.3)	3.0 (0.9–9.8)	3.4 (0.9–18.8)
	No	84 (84.0)	53 (94.6)	137 (87.8)			
	Total	100 (100)	56 (100)	156 (100)			
Young adults are equally vulnerable to dying	Yes	41 (40.6)	13 (23.6)	54 (34.6)	17.0 (0.8–33.1)	1.7 (1.0–2.9)	2.2 (1.0–5.0)
	No	60 (59.4)	42 (76.4)	102 (65.4)			
	Total	101 (100)	55 (100)	156 (100)			
Young adults show more signs	Yes	16 (15.8)	4 (7.3)	20 (12.8)	08.6 (−2.7–19.9)	2.2 (0.8–6.2)	2.4 (0.7–10.4)
	No	85 (84.2)	51 (92.7)	136 (87.2)			
	Total	101 (100)	55 (100)	156 (100)			
Young adults equally show signs	Yes	45 (45.5)	17 (31.5)	62 (40.5)	14.0 (−03.3–31.2)	1.4 (0.9–2.3)	1.8 (0.93.9)
	No	54 (54.5)	37 (68.5)	91 (59.5)			
	Total	99 (100)	54 (100)	153 (100)			
Young adults are more vulnerable to severe signs	Yes	19 (19.4)	5 (9.3)	24 (15.8)	10.1 (−02.3–22.6)	2.1 (0.8–5.3)	2.4 (0.8–8.6)
	No	79 (80.6)	49 (90.7)	128 (84.2)			
	Total	98 (100)	54 (100)	152 (100)			
Young adults equally show severe signs	Yes	46 (46.9)	16 (28.6)	62 (40.3)	18.4 (1.5–35.2)	1.6 (1.0–2.6)	2.2 (1.0–4.8)
	No	52 (53.1)	40 (71.4)	92 (59.7)			
	Total	98 (100)	56 (100)	154 (100)			

*Risk estimates conducted with males being the reference category; aR, attributable risk; RR, relative risk; OR, odds ratio; 95% CI, 95% confidence interval*.

### Perceptions of Risk and Race Among Ugandans

A majority of participants agreed that all races were at risk of COVID-19, however some participants thought that other races were more at risk than others. Amongst those who thought that COVID was a disease of “the whites,” a majority of these were males (32.4%, 33/102, 95% CI: 23.82–41.88) compared to 26.3% females (*n* = 15/57, 95% CI: 16.1–38.9). Furthermore, no significant differences were found between males (5.9%, 6/102) and females (5.3%, 3/57) toward agreement that the disease also affects Blacks. Furthermore, a majority of respondents stated that all races show severe signs of COVID-19 and of these, a majority were females (57.9, 33/57%,) compared to 49.5% (50/101) who were males (Table 5).

**Table 5:** Description of participant responses on race amongst male and female Ugandans.

**Parameter compared to race**	**Variable**	**Frequency (%) of respondents**			**Risk estimates**		
		**Male**	**Female**	**Total**	**aR**	**RR**	**OR**
Vulnerable	White	33 (32.4)	15 (26.3)	48 (30.2)	6.5	1.3	1
	All races	53 (52.0)	33 (57.9)	86 (54.1)	−4.9	0.9	0.7
	Blacks	6 (5.9)	3 (5.3)	9 (5.7)	−1.0	0.9	0.7
	Not Sure	10 (9.8)	6 (10.5)	16 (10.1)	−0.5	0.9	0.8
	Total	102 (100)	57 (100)	159 (100)			
Susceptibility to death	White	37 (36.6)	13 (23.2)	50 (31.8)	13.4	1.6	1
	All races	50 (49.5)	32 (57.1)	82 (52.2)	−7.6	0.9	0.5
	Blacks	5 (5.0)	2 (3.6)	7 (4.5)	1.4	1.4	0.9
	Not Sure	9 (8.9)	9 (16.1)	18 (11.5)	−7.2	0.6	0.4
	Total	101 (100)	56 (100)	157 (100)			
More signs and symptoms	White	34 (33.7)	15 (26.8)	49 (31.2)	6.7	1.3	1
	All races	54 (53.5)	31 (55.4)	85 (54.1)	−1.9	1.0	0.8
	Blacks	6 (5.9)	2 (3.6)	8 (5.1)	2.4	1.7	1.3
	Not Sure	7 (6.9)	8 (14.3)	15 (9.6)	−7.4	0.5	0.4
	Total	101 (100)	56 (100)	157 (100)			
Severe signs	White	38 (37.6)	16 (28.1)	54 (34.2)	1.0	1.3	1
	All races	50 (49.5)	33 (57.9)	83 (52.5)	−8.4	0.9	0.6
	Blacks	6 (5.9)	3 (5.3)	9 (5.7)	0.7	1.1	0.8
	Not Sure	7 (6.9)	5 (8.8)	12 (7.6)	−1.8	0.8	0.6
	Total	101 (100)	57 (100)	158 (100)			

*Risk estimates conducted using a 2 x k analysis. aR, attributable risk; RR, relative risk; OR, odds ratio; 95% CI, 95% confidence interval*.

## Discussion

In this study, a majority of study participants were young adults (64%, 103/161) and this was in agreement with statistical reports which have stated that a majority (51.3%) of Ugandans are in the age range of 15–64 years (42). The Uganda National Bureau of Statistics has classified age groups of Ugandans of adults using the 18–30 and 31–64 age groupings for adults (43), demonstrating the importance of young adults in epidemiological surveys. The study showed that a large proportion of males felt they were most vulnerable to COVID-19; however, these sentiments were not shared by women. Findings in the study demonstrate some disparities in COVID-19 risk perceptions at a time when COVID-19 cases are progressively increasing on the African continent (13). The differences in perception of vulnerability between men and women in our sample is concerning. The WHO has stated that women are at greater vulnerability in Africa (25), a risk perception not shared by our sample of Ugandan respondents (RR = 1.8, 95% CI: 1.14–2.8).

While both males and females recognize the importance of COVID-19 in their households, nearly half (52.9%, 54/102) of male respondents in our study perceived that they were more likely to die from COVID-19 than their female counterparts, while most women disagreed with this perception (RR = 1.8, 95% CI: 1.1–2.7). These findings in Uganda are worrying since a majority of males affected in Europe are elderly (34), and in this exploratory study, this was not the case. Furthermore, a majority of females disagreed with their male counterparts, demonstrating a level of superior knowledge; however, reasons for these disparities were not investigated by the current study. Perceptions of heightened male risk are likely to be influenced by online reports of more males dying than females, in Europe and China (32). It also appears to show an under-appreciation of the structural reasons why women are vulnerable to COVID-19, since they provide the most informal care in families, have limited economic opportunities, and less power in decision making (44, 45).

There is a need to develop novel strategies for communication of COVID-19 risk in Africa (5). The prognosis for COVID-19 patients in Asia and Europe appears to be affected mainly by sex, pre-existing health conditions, such as diabetes, cancers, and cardiovascular disease and being elderly (30), conditions which are also progressively rising in the African continent, despite poor prioritization of health service systems (46). However, women seek more health services than men (47). This may explain why women are able to mount stronger immunity than men (48); however, a young population in Africa is bound to have its own infection dynamics. Recent findings from Italy show no significant differences between genders (34). Symptomology in COVID-19 depends heavily on the immune status of a patient due to risky lifestyles like smoking and air pollution, and not necessarily on gender (33). In East African communities, household air pollution remains a public health threat especially in slums and rural communities where the use of firewood and charcoal continues to be routine (49). In Malaysia, knowledge has been shown to affect practice to minimize exposure to air pollutants (50), while a recent study in Uganda on COVID-19 has shown that knowledge affects practices promoted by the WHO against COVID-19 amongst market vendors (51).

Most male and female Ugandans in our sample believed that children below 18 years were less vulnerable to COVID-19 infections and that if they contracted the illness, they would be faced with a milder infection and that less would die from the disease. This is a common global perception that has been communicated by the media and many health agencies around the world in 2020 and is in agreement with studies that have shown a low infection rate in children (36). However, a significant number of women in this survey considered children to be equally vulnerable than adults and that children die more from the disease than adults (RR = 1.2, 95% CI: 0.9, 1.5). This may be due to maternal sentiments, communicated by women and not shared by men in Uganda, where men are often believed to show less empathy toward children (52).

The general opinion that elderly persons are more vulnerable to COVID-19 than young people is in agreement with recent epidemiological findings (42). Elderly individuals are known to be at high risk for COVID-19 (30). In general, women appear more knowledgeable than men on SARS-CoV-2; they may have a greater interest in health-related topics and show more health seeking behavior than men (51). Such misconceptions may have significant and far-reaching influence on health-seeking behavior (53).

It has been proposed that conception is functional and if people can solve problems within their existing conceptual environment, then the drive to change one's opinion becomes weak, although this does not help in solving a current problem (54). Thus, this theory of conceptual change is embedded in a set of epistemological assumptions that are far more generalizable than our application to misconceptions has exploited. These epistemological assumptions suggest that the basic problem of understanding cognitive development is to understand how the components of an individual's conceptual ecology interact and develop and how the conceptual ecology interacts with experience (55).

Of concern in this study is the perception among 30.2% (48/159) of respondents, particularly men, that COVID-19 is a “white-man's disease;” these feelings were strongest amongst males. These sentiments reflect the present disunified response against COVID-19 in East African states. In Tanzania and Burundi prayers are being promoted for people to seek divine intervention (17). Many believe that Africa will “be spared” COVID-19, since the disease originated in Asia before spreading to Europe (1), and following reports that malaria endemic regions would be protected, from generational exposure to chloroquine and hydroxychloroquine amongst Africans, since these drugs were reported to have some success in COVID-19 treatment (56, 57). That Ugandans perceive COVID-19 and infection of “whites” or the “other” is in direct contradiction of data coming from the USA and UK where Black, Asian, and minority ethnic (BAME) communities have been hit hard with infection.

Projections, from the WHO and others, indicate over 10 million cases of COVID-19 in Africa (8), and 3 million COVID-19 related deaths in the coming months of 2020 (11). Quantifying pandemic growth and assessment of interventions put in place (16) will not be straightforward in low- and middle- income settings faced with challenges of access to testing and reporting of cases and deaths. While many African nations have indeed learned from experiences of Ebola (15), SARS-CoV-2 is a far greater challenge. The virus itself and interventions that have been put in place are both new and difficult to manage long-term in LMICs. Disunity of policies across Sub-Saharan Africa (17, 18, 20, 21), will not enable disease containment necessary for economic and social recovery and will fuel sustained community transmission (3).

### Study Limitations

The study was conducted through an Online application, meaning that Ugandans without smart phones connected to the internet were not able to participate. Recruitment was achieved through sharing of the online link *via* social media and email platforms and data in this study was generally from the same age group i.e., 18–31 years. The sample size was small and further large-scale studies are needed to extend this exploratory study. Furthermore, results should be approached with caution until more large-scale studies are conducted which would include asymptomatic variables not investigated in the current study.

## Data Availability Statement

The datasets presented in this study can be found in online repositories. The names of the repository/repositories and accession number(s) can be found in the article/Supplementary Material.

## Ethics Statement

Expediated ethical approval was acquired from the Institutional Review Board of Kampala International University under ID: Nr.UG-REC-023/201914. Consent to participate was acquired through online acceptance to participate in the study.

## Author Contributions

KK and SW conceptualized the study. KK, SW, and FS designed the study. KK, FS, KM, GM, RS, IE, EA, RM, GN, HO, GZ, and JE conducted data acquisition. KK, EM, KB, MM, and SW conducted statistical analysis. KK, FW-S, EM, KB, MM, and SW conducted interpretation. KK, SW, and KB drafted the initial manuscript. KK, EM, KB, FS, MM, KM, GM, RS, FW-S, IE, EA, RM, GN, HO, GZ, JE, and SW revised the manuscript for intellectual content and approved the final version for publication. All authors agree to be accountable for all aspects of the work.

## Conflict of Interest

The authors declare that the research was conducted in the absence of any commercial or financial relationships that could be construed as a potential conflict of interest.

## Abbreviations

nCTF: National COVID-19 Task Force
WHO: World Health Organization
LMIC: Low Middle income Countries
BAME: Black, Asian, Minority Ethnic
CDC: Center for Disease Control.
