# Supplementary material for: Misconceptions on COVID-19 Risk Among Ugandan Men: Results From a Rapid Exploratory Survey, April 2020
Source: Front Public Health. 2020 Jul 28;8:416. doi: 10.3389/fpubh.2020.00416 (PMC7405654; doi:10.3389/fpubh.2020.00416)
Supplement: Supplementary file 1 [file Table_1.DOCX]

**Supplement File 1. Misconceptions on COVID-19 Risk among Ugandan Men. The questionnaire.**

What is your sex? Male=0, Female= 1; State your age?

**Section 1: Perceptions amongst males and females in Uganda on COVID-19 with sex**

1. Do you think males are more vulnerable than females to getting COVID-19 illness in the future? 0= no, 1 =yes
2. Do you think males and females are equally vulnerable to COVID-19 infection in Uganda? 0= no, 1 =yes
3. Do you think males are more likely to die from COVID-19 illness in the future than females in Uganda? 0= no, 1 =yes
4. Do you think males and females are equally vulnerable to dying from COVID-19 illness in the future? 0= no, 1 =yes
5. Do you think males are more vulnerable than females to showing signs and symptoms of COVID-19 illness in the future when infected in Uganda? 0= no, 1 =yes
6. Do you think males and females are equally vulnerable to showing signs and symptoms of COVID-19 illness in the future when infected? 0= no, 1 =yes
7. Do you think males are more vulnerable than females to showing severe signs and symptoms of COVID-19 illness in the future when infected in Uganda? 0= no, 1 =yes
8. Do you think males and females are equally vulnerable to showing severe signs and symptoms of COVID-19 illness in the future when infected in Uganda? 0= no, 1 =yes

**Section 2: Perceptions amongst males and females in Uganda on COVID-19 with age**

1. Do you think children are more vulnerable than adults to getting COVID-19 illness in the future? 0= no, 1 =yes
2. Do you think children and adults are equally vulnerable to COVID-19 infection in Uganda? 0= no, 1 =yes
3. Do you think adults are more likely to die from COVID-19 illness in the future than children in Uganda? 0= no, 1 =yes
4. Do you think adults and children are equally vulnerable to dying from COVID-19 illness in the future? 0= no, 1 =yes
5. Do you think adults are more vulnerable than children to showing signs and symptoms of COVID-19 illness in the future when infected? 0= no, 1 =yes
6. Do you think adults and children are equally vulnerable to showing signs and symptoms of COVID-19 illness in the future when infected in Uganda? 0= no, 1 =yes
7. Do you think adults are more vulnerable than children to showing severe signs and symptoms of COVID-19 illness in the future when infected? 0= no, 1 =yes
8. Do you think adults and children are equally vulnerable to showing severe signs and symptoms of COVID-19 illness in the future when infected in Uganda? 0= no, 1 =yes
9. Do you think young adults are more likely to die from COVID-19 illness in the future than the elderly people in Uganda? 0= no, 1 =yes
10. Do you think young adults and the elderly people are equally vulnerable to dying from COVID-19 illness in the future in Uganda?
11. Do you think young adults are more vulnerable than the elderly to showing signs and symptoms of COVID-19 illness in the future when infected? 0= no, 1 =yes
12. Do you think young adults and the elderly are equally vulnerable to showing signs and symptoms of COVID-19 illness in the future when infected in Uganda? 0= no, 1 =yes
13. Do you think young adults are more vulnerable than the elderly to showing severe signs and symptoms of COVID-19 illness in the future when infected in Uganda? 0= no, 1 =yes
14. Do you think young adults and the elderly are equally vulnerable to showing severe signs and symptoms of COVID-19 illness in the future when infected in Uganda? 0= no, 1 =yes

**Section 3: Perceptions amongst males and females in Uganda on COVID-19 with race**

1. Which of the following races is more vulnerable to getting COVID-19 illness in the future? 1= Whites, 2 =all, 3=Asians, 4= Blacks, 5= Not sure
2. Which of the following races is more likely to die from COVID-19 illness in the future? 1= Whites, 2 =all, 3=Asians, 4= Blacks, 5= Not sure
3. Which of the following races is more vulnerable to showing signs and symptoms of COVID-19 illness in the future when infected in Uganda? 1= Whites, 2 =all, 3=Asians, 4= Blacks, 5= Not sure
4. Which of the following races is more vulnerable to showing severe signs and symptoms of COVID-19 illness in the future when infected in Uganda? 1= Whites, 2 =all, 3=Asians, 4= Blacks, 5= Not sure

The Authors.
